# Chemical analysis, antibacterial and anti-inflammatory effect of *Achillea fragrantissima* essential oil growing wild in Egypt

**DOI:** 10.1186/s12906-024-04633-9

**Published:** 2024-11-07

**Authors:** Nashwa F. Tawfik, Nashwa El-Sayed, Shahenda Mahgoub, Mohamed T. Khazaal, Fatma A. Moharram

**Affiliations:** 1 Pharmacognosy Department, Faculty of Pharmacy, Helwan University, Ein Helwan, 11795 Egypt; 2 Department of Biochemistry and Molecular Biology, Faculty of Pharmacy, Helwan University, Ein Helwan, 11795 Egypt; 3 Botany and Microbiology Department, Faculty of Science, Helwan University, Ein Helwan, 11795 Egypt

**Keywords:** Antibacterial, Anti-inflammatory, Biofilm, Essential oils, Gas chromatography, *Achillea fragrantissima*

## Abstract

**Background:**

*Achillea fragrantissima* (*F. Asteraceae*) is traditionally used to treat skin infections and inflammation. The present work intended to prepare essential oils (EOs) from *A****.**** fragrantissima* aerial parts growing widely in Egypt and investigate its antibacterial activity against skin-related pathogens and in vitro cell-based anti-inflammatory activity.

**Methods:**

EOs of the fresh aerial parts were extracted by hydrodistillation (HD), microwave-assisted hydrodistillation (MAHD), and head-space (HS), while those of the dried ones were prepared by supercritical fluid (SF). The result EOs were analyzed using GC/MS. The antibacterial activity was evaluated alongside *Pseudomonas aeruginosa* ATCC 9027, *Escherichia coli* ATCC 8739, *Staphylococcus aureus* ATCC 25923, *Streptococcus pyogenes* ATCC 12344, *Clostridium perfringens* ATCC 13124 by agar diffusion, microwell dilution, and biofilm formation tests. The anti-inflammatory activity was evaluated by measuring tumor necrosis factor-alpha (TNF-α), interleukin 2 (IL-2), and 6 (IL-6) in lipopolysaccharides (LPS)- stimulated RAW 264.7 cells using ELISA assays in addition, expression of nitric oxide synthase (iNOS) was measured via western blot.

**Results:**

The SF method gave the highest EO yield (1.50 mL v/w). Oxygenated components constituted the highest percentage in the four methods, 84.14, 79.21, 73.29 and 33.57% in the HS, HD, MAHD, and SF, respectively. Moreover, variation in the amount of identified compounds was apparent; in HS EO *α-*thujone (29.37%), artemisia ketone (19.59%), and santolina alcohol (14.66%) are major components, while *α-*thujone (20.38%) and piperatone (12.09%) were significant in HD. Moreover, ( +)-spathulenol (12.22%) and piperatone (10.48%) were significant in MAHD, while piperatone (14.83%) and *β*-sitosterol (11.07%) were significant in SF EO. HD, MAHD, and SF EOs exhibited susceptibility against *P. aeruginosa* (IZ = 9–14 mm), *E. coli* (11–13 mm), and *C. perfringens* (IZ = 10–14 mm) in agar diffusion assay. MAHD EOs demonstrated potent growth inhibition (MICs = 0.25–2 mg/mL), followed by HD EOs (MICs = 13–52 mg/mL) to all tested microorganisms in well microdilution assay. Also, they exert MBC values equal to or higher than the MICs. Furthermore, SF EOs inhibited the biofilm formation of all tested microorganisms by 65.12—80.84%. Specifically, MAHD and HD EOs efficiently suppress the biofilm of *S. pyogenes* (77.87%) and *P. aeruginosa* (60. 29%), respectively. Ultimately, HD and SF EOs showed anti-inflammatory activity by suppressing the TNF-α, IL-2, and IL-6 release and iNOS expression in LPS-stimulated RAW 264.7 macrophages.

**Conclusion:**

*A****.**** fragrantissima* EO is rich in oxygenated volatile compounds with antibacterial and anti-inflammatory activities. It is encouraged as a bioactive agent for adjusting skin infections, though additional studies are essential for their safety in clinical settings.

**Supplementary Information:**

The online version contains supplementary material available at 10.1186/s12906-024-04633-9.

## Background

The skin of humans is always exposed to external factors, which makes the tissue of the skin among the most public infections. Skin infections can be caused by infection with different microbes, mainly bacterial and fungal, which are considered the most common due to temperature and moisture occasionally accompanying deprived hygiene [1]. So, the skin is a perfect environment for the growth of microbes because it is a rich source of nutrients, water, and high temperature. Commonly, there is a relationship between the normal microbial flora and the human host which protects it from infection by other pathogenic via location competition and the production of antimicrobial agents [2]. The last procedure results in cross-reactive antibodies, which are active against other aggressive microbes. Therefore, bacterial or fungal infection arises when the host and normal microorganisms are imbalanced [1]. The microorganisms can form a biofilm to protect themselves from outside distress by generating a promising environment, permitting microbial communication, improved virulence and nutrient breakdown, and supporting microbial progression and development. Thus, microorganisms have protection, sustainability, and survival against negative environmental conditions, immune systems, and antibiotics [3]. Formation of the biofilm by the skin’s indigenous microbiota can help inhibit its infection. The protection afforded by the indigenous microbiota is referred to as colonization resistance, and it is a significant skin defensive mechanism that prevents exogenous bacteria and fungi from attaching to the surface of the skin [4]. Generally, dressings and topical antimicrobial agents are routinely used to prevent skin infections [5], but they may be affected by pathogenic microorganisms. Moreover, the antibiotics presented are frequently ineffective due to infections with drug-resistant bacteria such as *P. aeruginosa* and *S. aureus* [6–8]. Moreover, anti-inflammatory infection treatment is challenging due to the heterogeneity of etiologic agents and complex immune interactions. Inflammation plays an essential role in the pathophysiology of the infection, being one of the main body defensive mechanisms. Anti-inflammatory agents have been used to treat infections as supportive agents to dismiss associated symptoms. Moreover, anti-inflammatory drugs act as host response modifiers and might play a critical role in infection management with extreme inflammation, avoiding injury to the organ [9]. Throughout the previous numerous decades, multiresistant bacteria and fungi have been considered an essential problem worldwide, and choosing a suitable treatment for skin infections is a challenge [10, 11]. Moreover, using pharmacological drugs to treat skin infections is expensive and not always effective. So, several researchers have been trying to find safe and effective antimicrobial agents for resistant microorganisms affecting skin infections. Natural products and traditional medicines are considered as one approach used for the treatment of skin infection, among which essential oils (EOs), which exhibited antimicrobial effects against multiresistant bacteria [1, 12–14] due to a broad spectrum of biocidal activity [15, 16], in addition, several EOs exhibits strong anti-inflammatory activity [17]. EOs are a hydrophobic, complex mixture of organic volatile components composed mainly of terpenoids, aliphatic, and aromatic compounds [18]; each class is further classified into oxygenated and non-oxygenated components [19]. The variability in chemical components of the EOs could be attributed to some factors, including the preparation technique, geographic bases, environmental factors, maturity stage, plant organ, and genetics [20, 21]. Numerous medicinal plant species are characterized by EO, among which plants belong to the genus *Achillea* [22]. The genus *Achillea* (F. Asteraceae), commonly known as Yarrow, comprises about 130 species of perennial plants, mainly growing in temperate regions in Europe and Asia [22]. Traditionally, *Achillea* species were used as tonic, anti-inflammatory, anti-spasmodic, carminative, diaphoretic, diuretic, and emmenagogic agents. They were also used for treating hemorrhage, pneumonia, rheumatic pain, and wound healing [22, 23]. *A. fragrantissima* (Forssk.) Sch.Bip (Syn. *Santolina fragrantissima* Forssk.) is an aromatic perennial plant growing widely in North Africa, the eastern Mediterranean coast, and the Middle East regions [24]. It is commonly known as Qaisoom in the Middle East [25], considered one of the most important traditionally used medicinal herbs in the Arabic region [26]***.*** It is traditionally used as a diuretic and to treat stomach and respiratory complaints. It is also used for skin infections, inflammation, diabetes, and wound healing [26–30]. Moreover, it is widely used in the folk medicine of Egypt for respiratory disease, eye infections, smallpox, fever, gastrointestinal disorders, diabetes, dysmenorrhea, and headache [29–31]. *A. fragrantissima* is rich in EOs, which attracts the attention of many authors to prepare it by different methods and evaluate its biological activities. *A. fragrantissima* EO -collected from different areas in Egypt- was prepared before by hydro distillation method (HD) [32–38], MAHD [34], and solid-phase microextraction (SPME) [38]. Moreover, antimicrobial [32, 34, 36, 39, 40], antioxidant [38], anti-inflammatory [41], and anticancer activities [37] were evaluated. To investigate the influence of extraction methods on the physical and chemical composition of *A. fragrantissima* EOs, we aimed to compare the volatile components of the *A. fragrantissima* growing wild in Egypt using four different extraction techniques. Additionally, we examined the potential of these EOs to inhibit pathogens associated with skin infections, their capacity to impede skin biofilm formation, and their cell-based anti-inflammatory activity.

## Material and methods

### Plant material

The aerial parts without flowers of *A. fragrantissima* (Forssk.) Sch. Bip was supplied from the Red Sea coastal region (Al-Ein Alsokhna, August 2022) in Cairo, Egypt. According to the local protected area’s rules for collecting and obeying Egypt’s collection legislation, the plant was supplied after the protected area authorities’ permission. The plant was identified by Prof. Abduo M Hamed, Plant Ecology Professor, Faculty of Science, Al-Azhar University, Cairo, Egypt. (sampling N0 37Afr 1/2022) and is placed at Pharmacognosy Department herbarium, Faculty of Pharmacy, Helwan University, Egypt.

### Essential oils extraction

#### Hydrodistillation (HD)

Fresh aerial parts were cut into small species (250 g), then covered with distilled water, and subjected to hydrodistillation for four h using Clevenger device [42].

#### Microwave-assisted hydrodistillation (MAHD)

It was accomplished by a microwave oven (CEM Corporation, Matthews, NC, USA), model (MARS 240/50, No. 907511, 1200 W), and worked at 2450 MHz. The small fresh aerial parts (200 g) were located in a flask containing 1L deionized water; then, the Clevenger device was put in the oven and a cooling system was used for condensation of the EO. The oven worked for one h at 800 W [42].

#### Supercritical fluid extraction (SF)

The extraction was done using supercritical carbon dioxide employing speed TM SFE-2/4, functional separations constructed with the USDA1-USA. 250 g shade-dried aerial parts were extracted at 40°C and 15.0 Mpa. Firstly, the device worked in a static mode followed by a dynamic one for one hour each, with a final total handing-out time of two hours. Absolute ethanol (0.2 mL/min/ flow rate) was added as a cosolvent to regulate the polarity and enhance solvating power. [42].

#### Dynamic head-space GC/MS analysis

It was done according to Hashemi et al. [43] and about 2 g of plant sample was in a glass vial (5 mL). A Shimadzu head-space sampler HS-20 joined to a Shimadzu GCMS-QP2020 (Kyoto, Japan) was working equipped with an Rtx-1MS column (30 m × 0.25 mm id. × 0.25 µm film thickness) (Restek, Bellefonte, PA, USA). The temperature of the oven and that of the sample and transfer lines were alleged at 80°C and 150°C, respectively. The conditions of head-space sampling are: the time of equilibration, pressurizing, and needle flush were fixed at 8.0, 2.0, and 5.0 min, respectively. The temperature of the column oven was adjusted at 45°C for two min, then increased to 300°C (5°C /min) and became constant for 5 min. Helium was used as a carrier gas (flow rate: 1.40 mL/min—a split ratio of 1: 25) was working. The pressure of APCI was established at 50 kPa, the ion source and interface temperature are 200°C, and 280°C, respectively.

The oil obtained EOs from the HD and MADH techniques was dried over anhydrous Na_2_SO_4_ Sigma-Aldrich, Saint Louis, Missouri, USA, and stored at 4 °C until GC/MS analysis. Moreover, the HD, MADH, and SF EOs percentages were stated in mL / 100 g of plant sample. [42].

### Gas chromatography-mass spectrometry (GC–MS) analysis of *A. fragrantissima* EOs

Shimadzu GCMS-QP2010 (Tokyo, Japan) was used. The column used is Rtx-1MS, 30 m × 0.25 mm i.d. × 0.25 µm film thickness, Restek, USA) with the split-splitless injector. The beginning temperature for the column was adjusted at 45°C for 2 min (isothermal), then programmed to 300 °C at a rate of 5°C/min and remained constant at 300°C for 5 min (isothermal). Moreover, the temperature of the flame ionization detector was 280°C. The carrier gas is helium, with a 1.41 mL/min flow rate. The ion source ( 200°C) and interface ( 280°C) temperature for MS analysis, and the electron ionization manner is 70 eV (scanning series is 35 to 500 amu). The oil sample (1 μL) was injected by split mode (1:15). Identification of the EO components was done by comparing Kovats’ retention indices (RI) with that of them with that of the *n*-alkanes standard (C_8_-C_28_) also, comparing their MS spectra with that mentioned in the NIST and Wiley database (similarity index > 90%) [44].

### Antibacterial activity

#### Materials

*P.aeruginosa* ATCC 9027, *E. coli* ATCC 8739, *S. aureus* ATCC 25923*, S.pyogenes* ATCC 12344*,* and* C. perfringens * ATCC 13124 were supplied from Research and Technology Institute, Micro laboratory (Vellore, Tamilnadu, India). Mueller–Hinton agar (MHA) and broth (MHB), dimethyl sulfoxide (DMSO, Sigma-Aldrich, Saint Louis, Missouri, USA), as well as the discs of antibiotic standard (6.0 mm, Oxoid, Thermo Fisher Scientific, USA).

#### Susceptibility test

The antibacterial effect of the investigated EOs was assessed by the agar well-diffusion method, [45, 46]. In brief, each reference strain (100 µL, 1 × 10^5^ CFU/mL) was spread evenly on the MHA medium. After solidification, 0.6 cm wells were created, and different concentrations of EOs (50 µL) were added individually to each well. After that, the plates were set in a refrigerator (30 min) and then incubated for 24 h at 37°C. However, in the case of *C. perfringens*, incubation was carried out anaerobically using an anaerobic jar. The antibacterial vulnerability of the EOs was measured by calculating the inhibition zone diameters (IZ) in mm. Conventional antibiotics were used as positive control comprising amikacin (AK, 30 μg/mL) as protein synthesis inhibitor, amoxicillin (AX, 25 μg/mL), and ampicillin/sulbactam (SAM, 20 μg/mL) as cell wall synthesis inhibitors, as well as norfloxacin (NOR, 10 μg/mL) and ofloxacin (OFX, 5 μg/mL) as DNA replication inhibitors. Sterile DMSO (10%) was used as a negative control.

#### Assessment of minimum inhibitory concentrations (MICs)

They were measured by broth microdilution assay [45, 47, 48]. Stock solutions were prepared by dissolving 52, 4, and 185 mg of HD, MAHD, and SF EOs, respectively, in 1 mL 10% DMSO. Subsequently, MHB (100 µL) was added to each well (2 to 12), while stock solutions (150 µL each) were added to the microtiter plates (first column). Two-fold dilution was carried out by transporting 100 µL from the 1^st^—11^th^ well. Next, to each well microbial culture (100 µL, OD_625nm_ = 0.2, 1 × 10^5^ CFU/mL) was added while the last one served as blank, followed by incubations of all plates (24 h at 37°C), except for *C. perfringens*, incubation was carried out anaerobically using an anaerobic jar. The absorbance measurements were carried out at λ_max_ 620 nm by an automated microplate reader (ChroMate 4300, USA), and they were graphically represented by Microsoft Excel version 2019. Statistical significance was measured using a *p*-value of < 0.05 in comparison to the control. The cell viability % was calculated using the formula (A_treated_/A_untreated_) × 100, where A is the absorbance at 620 nm, and the viability % of untreated cells was considered 100%.

#### Assessment of the minimum bactericidal concentration (MBC)

It was calculated using the dilution in broth method [45]. A liquots of 50 µL from all wells that showed no visible growth after 24 h of incubation at 37°C were spread on MHA plates and then incubated for 24 h at 37°C with anaerobic incubation for *C. perfringens* using an anaerobic jar. As the technique’s detection limit (LOD) is 10 cfu/mL, the absence of any growth on the plates indicated that the concentration was below this threshold. Consequently, the beginning concentration of 105 cfu/mL had been effectively decreased to below ten. Subsequently, the minimum concentration of EO capable of killing more than 99.99% of the present bacteria is assessed as the MBC. Three replicates of each trial were performed.

#### Anti-biofilm formation quantitative assay

It was assessed following Salem et al. [48] and Kang et al. [49], with few changes. Concisely, different concentrations of HD EO (0.1–52 mg/mL, MAHD EO (0.01–4 mg/mL), and SF EO (0.361–185 mg/mL) were added to 96-well polystyrene plates, which inoculated with 100 μL of each microbial culture (1 × 10^5^ CFU/mL) individually. Statically, the plates were incubated for 48 h at 37°C, with anaerobic incubation for *C. perfringens,* then washed two times with sterile PBS (pH 7.3), followed by air drying. The biofilms were fixed by adding 400 μL MeOH (15 min), then stained with crystal violet solution (1%,10 min). Lastly, the stained biofilms were solubilized by adding absolute EtOH to each well. The absorbance was measured at 630 nm by a microplate reader (ChroMate 4300, USA).

The results were expressed as the percentage of inhibition of biofilm formation using the following formula [(OD_control_-OD_sample_)/ (OD_control_)] × 100, where OD is the optical density measured at 630 nm and the untreated cells was used as a control.

### Anti-inflammatory activity

It was carried out on HD and SF samples since the MADH samples are finished in the analysis and antibacterial activity.

#### Cell culture and viability test

The murine RAW 264.7 monocyte/macrophage cells were delivered by the American Type Culture Collection (Tib 71) (Manassas, VA, USA). The cells were cultured as described earlier by Mahgoub et al. [50]. It is a famous cell line often used to screen natural products or other compounds for their bioactivity and predict their potential impact in vivo. This cell line cytokine response can be considered to reflect the potential human response.

#### In vitro MTT-based toxicology assay

(Sigma-Aldrich, Saint Louis, Missouri, USA) kit was used to determine cell viability as defined by the manufacturer. RAW cell viability was assessed by comparing the treated cells to the non-treated control. The sample concentration necessary for the inhibition of 50% cell growth (IC_50_) was estimated from the dose–response curve using nonlinear regression analysis. For determination of the proinflammatory markers, cells were treated with ¼ IC_50_ of HD and SF EOs for two h, followed by induction of inflammation using 1.0 μg/mL of LPS. 24 h later, the cells were collected for ELISA analysis in cell lysate.

#### Enzymes Linked Immunosorbent Assay (ELISA)

ELISA was applied to determine TNF-α (My BioSource Mouse TNF alpha ELISA Kit: Catalog #: MBS825075), IL-2 (Cusabio® Mouse IL-2 ELISA Kit: Catalog # CSB E04627m) and IL-6 (RayBio® Mouse IL-6 ELISA Kit: Catalog #: ELM-IL6-CL).

#### Western blot for iNOS

Cell protein lysates were prepared via agitation for 30 min (4°C) using Lysis buffer (50 mM Tris–HCl, pH 8.0, 150 mM NaCl, 0.1% Triton X-100 0.5% sodium deoxycholate 0.1% SDS, one mM sodium orthovanadate, one mM NaF, and Protease inhibitors) in a precooled centrifuge. To determine the protein concentration, 10–20 μL of lysate was used to carry out the DC (Bio-Rad) protein assay developed from Lowry’s protocol for protein measurement. An equal concentration of each sample protein was added to 2 × Laemmli sample buffer (4% SDS, 10% 2-mercaptoethanol, 20% glycerol, 0.004% bromophenol blue and 0.125 M Tris–HCl) and heated for 5 min at 95°C then loaded on a mini SDS-PAGE gel along with molecular weight markers. Afterwards, the blots were transferred to a nitrocellulose membrane for 30 min. Each membrane was blocked with 3% BSA in TBST (Tris-buffered saline with Tween 20) at RT for one h. After that, it was incubated overnight with the primary antibodies (NOS2 Antibody (N-20)) rabbit polyclonal antibody, which was provided from Santa Cruz Biotechnology (Santa Cruz, CA, USA) or beta Actin Monoclonal Antibody (ThermoFisher Scientific, MA, USA, Cat #: AM4302), then incubated with secondary HRP-linked antibody. Chemiluminescent substrate (PerkinElmer, USA) was applied to the immunoblots. The chemiluminescence signals were then taken using a CCD camera-based (Chemi Doc imager, Biorad, USA) and analyzed by ImageLab (Biorad). Cell lysates stimulated with LPS were employed as a positive control.

### Statistical analysis

Statistical methods were applied by ANOVA using GraphPad Prism 5 (GraphPad Inc, CA, USA), and Duncan’s post hoc test using IBM SPSS version 20 software was performed to compare the reduction of optical density (OD) through viability and biofilm percentage accompanying each investigated sample to that of untreated control. The mean inhibitory concentration (IC_50_) was obtained from the dose–response curves by GraphPad Prism 5. All data are stated as mean ± SD.

## Results

Choosing a suitable method for extracting the EO from aromatic plants is tedious based on some factors [51]. The HD represents the most used method, but it has some drawbacks due to the isomerization, saponification, and /or polymerization of the most sensitive components [52]. On the other hand, the MAHD and SF methods represent the environmentally friend and green methods and give high-quality EOs with slight decomposition in little time [53]. Moreover, the SF technique is advantageous because of its diffusion coefficient, high oil yield, and low viscosity. In addition, the HS-SPME tools are straightforward and efficient, need small plant samples, and can be reused in an environmentally friendly manner [48]. Therefore, it is clear that each technique differs in its essential attitude and conduction, which then affects the oil’s yield and physical and chemical properties. In this work, the EO of *A. fragrantissima* aerial parts growing wild in Egypt was extracted using HD, MAHD, and HS-SPME to study the effect of the technique used on the EO’s yield and volatile components. HD method is the most used technique for the extraction of *A. fragrantissima* aerial parts growing in Egypt [30–38], while MAHD [34] and SPME [38] were used once before but this is the first time to prepare the EO by HS and SF as well as compare the four methods with each other. Our result shows that the extraction method affects the oil yield being 1.0, 0.5, and 1.5 mL v/w in HD, MAHD, and SF, respectively, as well as the color is yellow in HD, orange in SF and pale yellow in MAHD, on the other hand, in case HS-SPME method the oil was unrecoverable.

### GC/MS analysis *A. fragrantissima*

Our results show that the composition of the EO is qualitatively and quantitatively affected by the technique used to extract *A. fragrantissima.* A total number of 14, 21, 26, and 17 components were identified in the case of HS, HD, MAHD, and SF, respectively, constituting 93.87% (HS), 84.74% (HD), and 79.82% (MAHD) and 57.23% (SF) (Table 1, Fig. S1-S4). Variation in the amount and type of the identified compounds was apparent; in HS EO *α*-thujone (29.37%), artemisia ketone (19.59%), santolina alcohol (14.66%) and *β*-thujone (10.09%) represent the highest percentage while in case of HD, *α*-thujone (20.38%) represents the highest percentage as in HS followed by piperatone (12.09%) santolina alcohol (9.00%). Moreover, in the case of MAHD, ( +)-spathulenol (12.22%) and piperatone (10.48%) are the major components, as well as piperatone (14.83%) and *β*-sitosterol (11.07%) are major in SF EO. In addition, the extraction method can affect the chemical class percentage as exposed from the difference in the result of *A. fragrantissima* EO, wherever the oxygenated components in general represent the highest percentage in comparison to non-oxygenated components being 84.14, 79.21, 72.58 and 33.57% in case of HS, HD, MAHD, and SF EOs, respectively, while, the non-oxygenated components percentage being 9.73% (HS), 5.53% (HD), 6.48% (MAHD), and zero% (SF). Moreover, the additional motivating difference was detected in the chemical class of the EO components since the composition of oxygenated monoterpene (OM) represents the highest percentage in all methods, being (84.14, 71.25, 45.29, and 23.37%) HS-SPME, HD, MAHD, and SF, respectively. In addition, it was observed that oxygenated sesquiterpenes in MAHD (24.85%) represent the highest percentage, while it is absent in the case of HS EO. For monoterpene hydrocarbons (MH), a large percentage was observed in HS (8.40%), while nearly the same percentage of sesquiterpene hydrocarbons was observed in HD and MAHD (≈ 5.5).

**Table 1:** Identified chemical composition of *A. fragrantissima* aerial parts essential oil extracted by HS, HD, MAHD, SF*

Peak	R_t_	Compound	MF	RI_Exp_	RI_Lit_	Content Percentage				
						**HS**	**HD**	**MADH**		**SF**
**1**	6.906	Santolina triene	C_10_H_16_	900	901	6.96	-	-		-
**2**	7.429	2-Carene	C_10_H_16_	919	983	0.30	-	-		-
	7.638	*α*-Pinene	C_10_H_16_	927	927	1.00	-	-		-
**4**	8.706	2-Thujene	C_10_H_16_	966	968	0.14	-	-		-
**5**	9.445	Yomogi alcohol	C_10_H_18_O	987	987	1.14	5.30	2.89		-
**6**	10.113	*p*- Cymene	C_10_H_14_	1015	1015	0.79	-	-		-
**7**	10.335	Eucalyptol	C_10_H_18_O	1022	1022	5.30	-	-		-
**8**	10.571	Santolina alcohol	C_10_H_18_O	1030	1029	**14.66**	**9.00**	**6.55**		-
**9**	11.246	Artemisia ketone	C_10_H_16_O	1051	1049	**19.59**	2.37	0.79		-
**10**	12.071	Artemisia alcohol	C_10_H_18_O	1069	1069	0.52	1.28	0.93		-
**11**	12.500	*α*-Thujone	C_10_H_16_O	1083	1082	**29.37**	**20.38**	**8.87**		3.56
**12**	12.787	*β*-Thujone	C_10_H_16_O	1092	1092	**10.09**	6.21	2.64		1.54
**13**	12.855	Chrysanthenone	C_10_H_14_O	1095	1094	-	1.23	1.13		-
**14**	13.095	4-Thujanol	C_10_H_18_O	1102	1100	-	0.54			-
**15**	13.426	Camphor	C_10_H_16_O	1113	1113	-	2.68	1.80		-
**16**	13.603	*Trans-*Sabinol		1119	1117	-	3.68	3.04		-
**17**	14.350	Borneol	C_10_H_18_O	1143	1143	-	-	0.96		-
**18**	14.364	Isoborneol	C_10_H_18_O	1144	1144	-	1.13	-		-
**19**	14.688	Artemisia acetate	C_12_H_20_O_2_	1162	1161	3.47	1.67	2.19		
**20**	16.334	*α*-Terpineol	C_10_H_18_O	1208	1208	-	1.08	0.96		
**21**	16.698	Piperitone	C_10_H_16_O	1221	1221	-	**12.09**	**10.48**		**14.83**
**22**	17.192	*α*-Fenchyl acetate	C_12_H_20_O_2_	1236	1240	-				1.58
**23**	18.110	*Trans*-sabinyl acetate)	C_10_H_18_O_2_	1269	1270	-	2.61	2.06		-
**24**	18.285	Cumic alcohol	C_10_H_14_O	1275	1275	-	0.64	-		-
**25**	20.065	Ethyl cinnamate (*Z*)	C_11_H_12_O_2_	1346	1338	-	-	0.75		-
**26**	20.851	Methyl eugenol	C_11_H_14_O_2_	1366	1366	-	-	0.57		-
**27**	22.439	Massoilactone	C_10_H_16_O_2_	1425	1433	-	-	-		0.95
**28**	22.454	(*E*)-Ethyl cinnamate	C_11_H_12_O_2_	1426	1426	-	-	1.12		-
**29**	23.155	Acenaphthene	C_12_H_10_	1453	1455	-	-	0.79		-
**30**	23.486	Germacrene D	C_15_H_24_	1474	1474	0.54	4.92	5.19		-
**31**	23.937	*β*-Cyclogermacrene	C_15_H_24_	1483	1483	-	0.61	0.50		
**32**	25.469	Nerolidol	C_15_H_26_O	1555	1551	-	-	1.31		-
**33**	25.732	( +)-Spathulenol	C_15_H_24_O	1568	1568	-	6.00	**12.22**		2.86
**34**	26.075	Salvial-4(14)-en-1-one	C_15_H_24_O	1586	1584	-	0.79	1.16		-
**35**	26.531	*β*-Copaen-4-*α*-ol	C_15_H_24_O	1588	1606	-	-	2.85		0.67
**36**	26.904	(*E*)-methyl jasmonate	C_13_H_20_O_3_	1619	1612	-	-	-		0.91
**37**	27.255	Alloaromadendrene oxide	C_15_H_24_O	1632	1622	-	-	-		0.91
**38**	27.467	Viridiflorol	C_15_H_26_O	1639	1636	-	-	7.31		2.67
**39**	27.535	*α*-Cadinol	C_15_H_26_O	1642	1642	-	0.53	-		-
**40**	28.216	Germacra-4(15),5,10(14)-trien-1α-ol	C_15_H_24_O	1680	1665	-	-	-		2.06
**41**	28.839	Isospathulenol	C_15_H_24_O	1667	1687	-	-	-		1.03
**42**	34.355	*n*-Hexadecanoic acid	C_16_H_32_O_2_	1940	1940	-	-	0.76		2.59
**43**	37.375	Phytol	C_20_H_40_O	2094	2096	-	-	-		2.38
**44**	43.908	Behenic alcohol	C_22_H_46_O	2467	2468	-	-	-		2.74
**45**	47.081	*n*-Tetracosanol-1	C_24_H_50_O	2670	2650	-	-	-		4.88
**46**	55.763	*β*-Sitosterol	C_29_H_50_O	3243	3220	-	-	-		**11.07**
						**Content Percentage**				
						**HS**	**HD**		**MADH**	**SF**
**Total identified compounds**						**93.87**	**84.74**		**79.82**	**57.23**
**Non-oxygenated compounds**						9.73	5.53		6.48	0.00
	Monoterpene hydrocarbons (MH)					8.40	0.00		0.00	0.00
	Sesquiterpene hydrocarbons (SH)					0.54	5.53		5.69	0.00
	Aromatic hydrocarbons (ArH)					0.79	0.00		0.79	0.00
**Oxygenated compounds**						84.14	79.21		72.58	33.57
	Oxygenated monoterpenes (OM)					84.14	71.25		45.29	23.37
	Oxygenated sesquiterpenes (OS)					0.00	7.32		24.85	10.2
	Oxygenated aromatic (OAr)					0.00	0.64		1.87	0.00
	Phenylpropanoid (PP)					0.00	0.00		0.57	0.00
**Miscellaneous compounds**						0.00	0.00		0.76	23.66

^*^HD hydrodistillation, MAHD Microwave-assisted hydrodistillation, SF Supercritical fluid extraction

### In vitro antibacterial activity

It was evaluated against five common skin pathogens, including the gram-negative bacteria *P. aeruginosa* and *E. coli,* and the three gram-positive bacteria *S. aureus*, *S. pyogenes,* and* C. perfringens.* The evaluation involved measuring the IZ in the agar well-diffusion assay, MICs in the broth microdilution test, and assessing the inhibition of biofilm development using the crystal violet test.

#### Agar diffusion assay

As shown from the result in Table 2, the susceptibility of *P. aeruginosa* ATCC 9027, *E. coli* ATCC 8739, and *C. perfringens* ATCC 13124 to the HD, MAHD, and SF-EOs with more or less the same IZ ranged from 10 to 14 mm.

**Table 2:** Inhibition zone (IZ) of the *A. fragrantissima* aerial parts extracted essential oils by HD, MAHD, SF

Samples	HD* µg/mL				MADH* µg/mL					SF* µg/mL				AK 30	AX 25	NOR 10	OFX 5	SAM 20
	**1000**	**500**	**250**	**100**	**1000**	**500**	**250**	**100**	**1000**		**500**	**250**	**100**					
Gram‑negative bacteria																		
* P. aeruginosa ATCC 9027*	13 ± 1	12 ± 1	10 ± 1	10 ± 1	14 ± 1	11 ± 1	11 ± 0	9 ± 1	14 ± 1		11 ± 0	10 ± 1	9 ± 1	21	-	30	20	-
* E. coli ATCC 8739*	13 ± 0	12 ± 1	11 ± 1	11 ± 1	13 ± 0	13 ± 0	13 ± 0	13 ± 0	13 ± 1		12 ± 1	11 ± 1	11 ± 1	22	-	30	29	19
Gram‑Positive bacteria																		
* S. aureus ATCC 25923*	**-**	**-**	**-**	-	**-**	**-**	**-**	**-**	**-**		**-**	**-**	**-**	22	21	25	27	24
* S. pyogenes ATCC 12344*	**-**	**-**	**-**	-	**-**	**-**	**-**	**-**	**-**		**-**	**-**	**-**	22	21	25	27	24
* C. perfringens ATCC 13124*	14 ± 1	13 ± 1	12 ± 1	12 ± 1	13 ± 1	12 ± 1	11 ± 1	11 ± 1	12 ± 1		12 ± 1	12 ± 1	10 ± 1	23	18	34	30	11

Results were stated as mean (*n* = 3) with SD ± 1. (-); not detected, amikacin, (AK 30); amoxicillin,(AX 25); norfloxacin, (NOR 10); ofloxacin, (OFX 5); ampicillin/sulbactam,(SAM 20); * HD (hydrodistillation); MAHD (microwave-assisted hydro-distillation); SF ( supercritical fluid)

 On the other hand, no susceptibility was observed for *S. aureus* ATCC 25923and *S. pyogenes* ATCC 12344 toward tested EO samples. The inhibitory effect strength was assumed in comparison to reference antibiotic discs. The relatively low IZ measurements could be attributed to the low diffusion rate of the EOs in the agar medium. Therefore, further investigation was conducted by measuring MICs to assess the inhibitory effects of the EOs more comprehensively.

#### Well microdilution assay for MIC and MBC

**Fig. 1:**
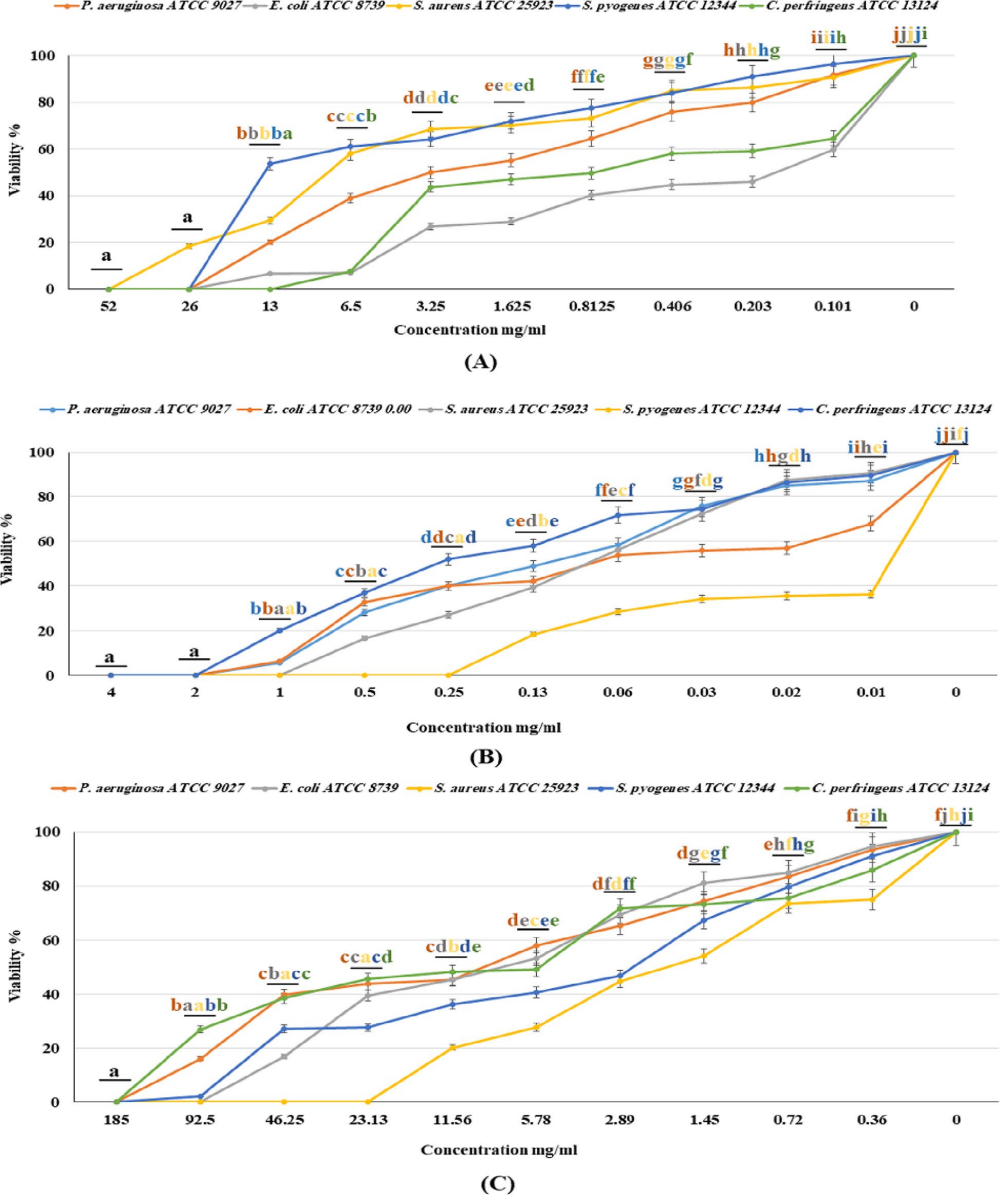
Dose–response effect of *A. fragrantissima* aerial parts HD EO (**A**); MADH EO (**B**); SF EO (**C**), on viability percentage of the skin pathogens in broth dilution assay. Error bars represent SD from the means (n = 3). Different letters of the means are significantly different (*p* < 0.000001) using Duncan’s post hoc test

The results of MIC (Table 3, Fig. 1) displayed the promising capability of the investigated oil sample to effectively decrease the growth of the particular skin infection bacterial strains, exhibiting variable potency levels. Interestingly, the MAHD EO showed more potent activity compared to HD and SF EOs. It exerted potent growth inhibition against *S. pyogenes* ATCC 12344 (MIC = 0.25 mg/mL), *S. aureus* ATCC 25923 (MIC = 1 mg/mL) and demonstrated equivalent inhibition activity against *P. aeruginosa* ATCC 9027, *E*. *coli* ATCC 9739 and *C. perfringens* ATCC 13124 (MICs = 2 mg/mL). Meanwhile, the HDEO displayed inhibition activity specifically against *C. perfringens* ATCC 13124*,* with MIC of 13 mg/mL. Additionally, SF EO showed moderate activity against *S. aureus* ATCC 25923 (MIC = 23.13 mg/mL) in comparison to the standard drug (1.0 mg/mL, chloramphenicol).

**Table 3:** MICs and MBCs of the tested EOs extracted from *A. fragrantissima* aerial parts by HD, MADH, and SF methods

Samples	HD* mg/mL	MADH* mg/mL	SF* mg/mL	C 1 mg/ml
Gram‑negative bacteria				
* P. aeruginosa ATCC 9027*	26 (26)	2.0 (4.0)	185 (185)	50
* E. coli ATCC 8739*	26 (26)	2.0 (4.0)	92.5 (185)	50
Gram‑positive bacteria				
* S. aureus ATCC 25923*	52 (52)	1.0 (2.0)	23.13 (46.25)	˃100
* S. pyogenes ATCC 12344*	26 (52)	0.25 (0.5)	185 (185)	˃100
* C. perfringens ATCC 13124*	13 (26)	2.0 (2.0)	185 (185)	˃100

Values not in brackets correspond to MICs, while those in brackets to MBCs)

***C*** Chloramphenicol, *P-value* * < 0.000001

^*^HD Hydro distillation, MAHD Microwave-assisted hydrodistillation, *SF* Supercritical fluid extraction

The MBC values were equal to or higher than the MICs for the tested EOs, and considerable variations were observed among strains. Table 3 presents the recorded values for MBCs (mg/mL), which ranged from 26 to 52 for HD EO, 0.5 to 4 for MAHD EO, and 46.25 to 185 for SF EO. The remarkable effectiveness of MAHD EO is indicated by its low MIC and MBC values. Our result is that since the MBC is equal to or higher than MIC, we expect the bactericidal effect for the extracted EOs [45].

#### Biofilm formation test

**Fig. 2:**
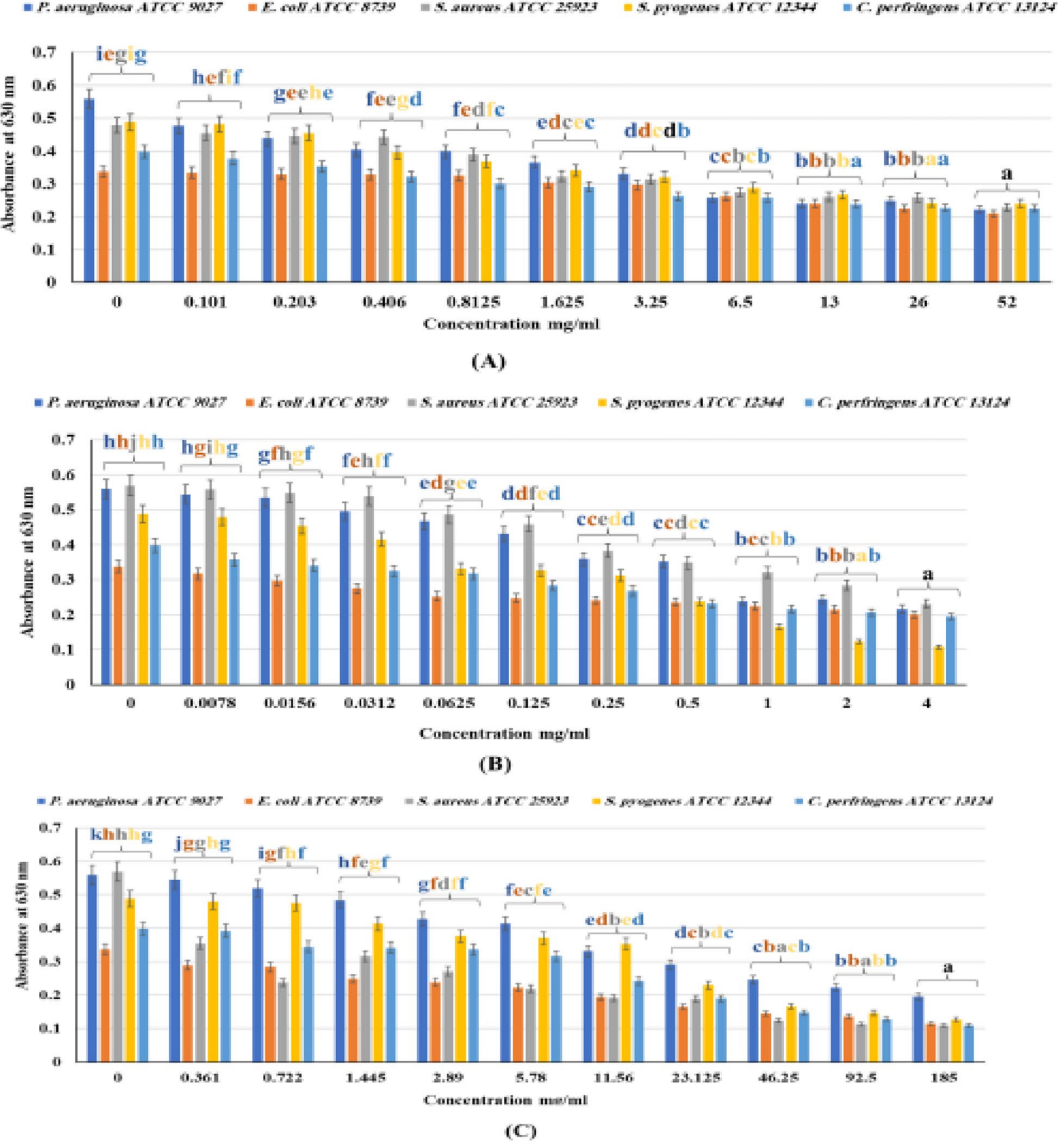
Dose–response effect of *A. fragrantissima* aerial parts HD EO (**A**); MADH EO (**B**); SF EO (**C**), on selected skin infection pathogens in biofilm formation test with concentration value “0” as a control. Error bars represent SD from the means (*n* = 3). Means with Different letters of the mean are significantly different (*p* < 0.000001) using Duncan’s post hoc

The results (Tables 4, 5, 6, Fig. 2) displayed that the inhibition percentages of the bacterial strains biofilm were significantly (*p* < 0.001) strengthened by increasing EO concentration. It is noteworthy that MAHD EO exhibits greater potency against *S. pyogenes* ATCC 12344. In contrast, SF EO positively inhibits biofilm formation, with percentages ranging from 65.12 to 80.84 for all tested strains. Specifically, the highest biofilm inhibition percentage, 80.84, 80.14, and 78.21%, was observed for *S. aureus* by the SF EO at 185, 92.5, and 46. 21 mg/mL, respectively. Additionally, the MAHD EO achieved a percentage of biofilm inhibition of 77.87% at a concentration of 4 mg/mL for *S. pyogenes*. SF EO also demonstrated potent biofilm inhibitory activity at 73.98% and 72.61% against *S. pyogenes* and *C. perfringens*, respectively, at the maximum tested doses. Moreover, HD EO displayed a biofilm inhibition percentage of 60.09% for *P. aeruginosa* at 185 mg/mL.

**Table 4:** Biofilm inhibition % of the HD-extracted essential oils from *A. fragrantissima* aerial parts

Sample	Conc. mg/mL*									
	**52**	**26**	**13**	**6.5**	**3.25**	**1.63**	**0.081**	**0.41**	**0.2**	**0.1**
Gram‑negative bacteria										
*P.aeruginosa ATCC 9027*	60.29	55.46	56.89	53.85	40.79	34.53	28.62	27.73	21.65	14.85
*E. coli ATCC 8739*	37.98	33.23	28.49	22.26	12.17	10.09	3.56	2.37	2.08	0.89
Gram‑positive bacteria										
*S.aureus ATCC 25923*	52.40	45.93	45.51	42.80	34.45	32.57	18.79	7.52	6.89	5.01
*S. pyogenes ATCC 12344*	50.61	50.41	45.29	40.78	34.22	29.92	24.39	18.85	6.76	1.43
*C. perfringens ATCC 13124*	43.47	42.96	39.95	35.18	34.17	26.88	24.37	19.10	11.31	5.03

*P-value* * < 0.000001

**Table 5:** Biofilm inhibition % of the MADH extracted essential oils from *A. fragrantissima* aerial parts

Samples	Conc. mg/mL*									
	**4**	**2**	**1**	**0.5**	**0.25**	**0.13**	**0.06**	**0.03**	**0.02**	**0.01**
Gram‑negative bacteria										
*P. aeruginosa ATCC 9027*	61.18	56.17	57.25	37.03	35.78	22.90	16.46	11.45	4.47	2.68
*E. coli ATCC 8739*	40.36	36.20	33.23	29.97	28.49	26.41	24.93	18.40	11.87	5.93
Gram‑positive bacteria										
*S. aureus ATCC 25923*	59.40	50.09	43.59	38.84	32.69	19.33	14.59	5.27	3.69	2.11
*S. pyogenes ATCC 12344*	77.87	74.59	65.98	51.23	36.07	33.20	32.17	14.96	7.17	1.84
*C. perfringens ATCC 13124*	51.01	47.99	45.73	41.71	32.66	28.89	20.35	18.34	14.32	10.30

*P-value* * < 0.000001

**Table 6:** Biofilm inhibition % of the SF extracted essential oils from *A. fragrantissima* aerial parts

Samples	Conc. mg/mL*									
	**185**	**92.5**	**46.25**	**23.125**	**11.56**	**5.78**	**2.89**	**1.445**	**0.722**	**0.361**
Gram‑negative bacteria										
*P.aeruginosa ATCC 9027*	65.12	60.11	55.99	47.94	40.79	25.94	23.61	13.42	7.16	2.68
*E. coli ATCC 8739*	66.17	59.64	57.27	51.04	42.43	33.83	29.38	26.11	15.73	14.24
Gram‑positive bacteria										
*S. aureus ATCC 25923*	80.84	80.14	78.21	66.96	66.43	61.51	52.37	44.46	58.35	37.61
*S. pyogenes ATCC 12344*	73.98	70.08	65.98	53.07	27.66	23.98	22.95	15.16	2.66	1.84
*C.perfringens ATCC 13124*	72.61	67.84	63.07	52.51	39.20	20.60	15.58	14.32	13.57	1.51

*P-value* * < 0.000001

### Anti-inflammatory studies

#### SF and HD EOs inhibit LPS-stimulated production of proinflammatory cytokines in RAW 264.7 cells

**Fig. 3:**
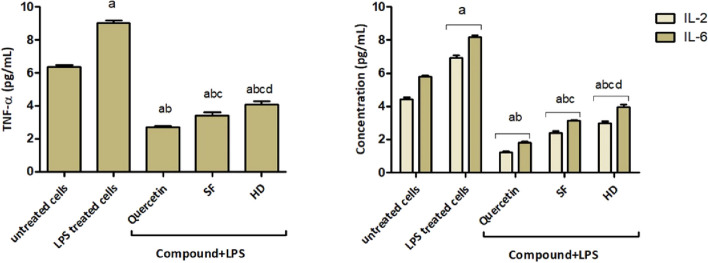
Effect of SF and HD EOs on TNF-α, IL-2, and IL-6 production by LPS-activated RAW 264.7. The results are stated as the means ± SD of three replicates. a, b, c & d, significant (*p* < 0.05) compared to untreated cells, LPS-only treated cells, quercetin, or SF

TNF-α, IL-2, and 6 represent the main proinflammatory cytokines in reaction to LPS. Initially, an MTT assessment was used to estimate the effect of SF and HD EOs on RAW 264.7 macrophage viability. IC_50_ values were calculated, and RAW 264.7 cell viability with these samples was determined to measure the sub-cytotoxic concentrations required to achieve the anti-inflammatory assays. Then, the cells were pre-treated with the dosages = (¼ IC_50_) of SF, HD EOs, or quercetin for two h, followed by treatment for 24 h with LPS (1 μg/mL). The results displayed that both SF and HD EOs significantly decreased the TNF-α, IL-2, and six productions by LPS-activated cells. (Fig. 3). Furthermore, our results indicated that SF EOs caused a significant decline in the determined proinflammatory cytokines, compared to LPS alone treated cells.

#### SF and HD EOs suppress iNOS expression in LPS-stimulated RAW 264.7 cells

**Fig. 4:**
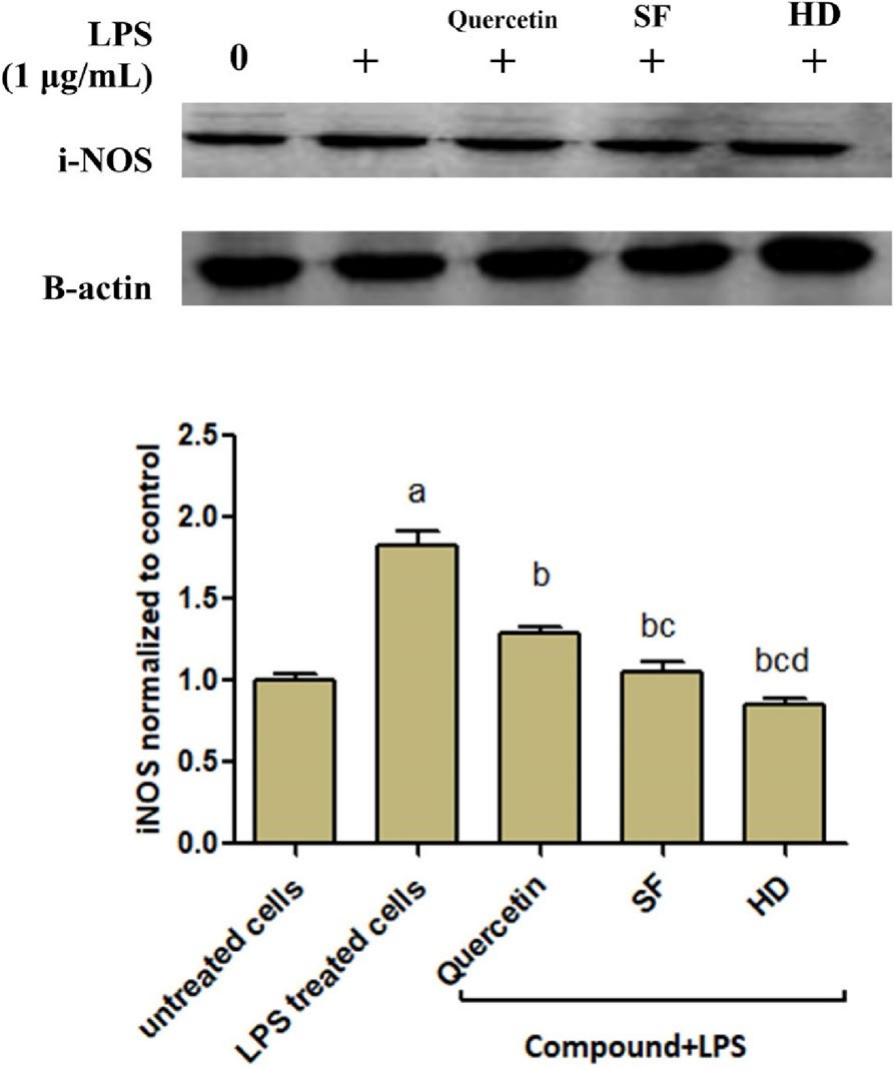
Effects of SF and HD on the iNOS production by LPS-activated RAW 264.7 cells Cells were incubated with quercetin, SF, or HD EOs for two h followed by stimulation with LPS (1 μg/mL,24 h). The iNOS expression was estimated by western blot. Results represent the means ± SD of three distinct experiments. a, b, c, or d, significant (*p* < 0.05) compared to untreated cells, LPS-only treated cells, quercetin, or SF

For investigation of the effect of SF and HD EOs on LPS-induced iNOS expression, RAW 264.7 cells were pre-treated with quercetin, SF, or HD- EOs at their non-cytotoxic dosages (¼ IC50) for two h. Then, they were treated with LPS (1 μg/mL, 24 h). As displayed in Fig. 4, SF and HD-24 h reduced the LPS-induced expression of iNOS compared to LPS-alone treated cells as measured by western blot.

## Discussion

The EO of *A. fragrantissima* was prepared using four methods to compare the effect of the extraction technique on the physical and chemical properties of the EOs. In the current study, our results revealed that the SF technique gave a higher yield for EO than the other two techniques (HD and MAHD) because, in the case of SF, supercritical CO_2_ (SCC) was used as an extraction solvent, which is categorized as a solvent posses low viscosity and surface tension, thus enhance the penetration rate, increasing the ability of extraction, and yield of the oil [54]. Moreover, its solvation power allows the co-solubilization of some fatty components, which provides the dark color and semi-solid properties for SF. Accordingly, the obtained EOs were subjected to GC/ MS analysis for qualitative and quantitative identification of their components. The results showed that volatile components differed amongst the extraction techniques, which may be responsible for the variances in plant material condition, applied pressure and temperature, and extraction time. In SF, dried plant material was extracted by SCC; in the case of HD, the fresh sample was boiled in water, while in MAHD, a microwave oven was used, but in HS, the fresh sample was heated till EO volatilization [42, 55, 56] which explain the difference in the obtained results.

On the other hand, even though the fresh sample was used in the HD, MAHD, and HS, temperature and extraction time variation significantly pretentious the extracted volatile components’ stability and percentage. Moreover, the solvent power used in the extraction acting an additional critical factor. In SF, SCC performs as a lipophilic solvent but with the benefit of adaptable selectivity, so fatty alcohol, *n*-hexadecanoic acid, and *β*-sitosterol were detected only in SF EOs. Also, in the case of HD, the water vapor penetration inside the plant sample improves the extraction of the low molecular weight oxygenated and non-oxygenated compounds than that of high molecular weight due to the long extraction time and high temperature [57, 58]. However, in the case of MAHD, the short exposure time allowed the evaporation of both low and high molecular weight compounds. Finally, the HS method is a comparative state of the art technique that is planned to produce volatile components with different boiling points without producing artifacts [59]. By comparing the obtained results with the previously reported for *A. fragrantissima* EO, collected from different areas in Egypt and prepared by HD [32–38], MAHD [34], and SPME EOs [38], we found that the reported results are consistent with our results, which revealed that oxygenated monoterpenes were the major constituents. In all previously reported data, there are deviations in the number and percentage of the identified compounds, which may be due to several factors, such as environmental complaints and genetic variants. Besides, the time of drying and collection, as well as the temperature, may affect the EOs both qualitatively and quantitatively. Additionally, *A*. *fragrantissima* is a perennial shrub, and it was reported that plant age may exert an essential factor in EO’s chemical components [60, 61].

Regarding the impact of the prepared EOs on skin infection, the antibacterial activity was estimated against five public skin pathogens, *viz*, *P. aeruginosa, E. coli, S. aureus, S. pyogenes, and C. perfringens*. They were designated based on their availability and pathogenic history of skin infection. For example, *S. aureus* is a commensal pathogen frequently found on healthy individuals’ skin and mucous membranes [62]. It is responsible for more than 80 % of all human skin and soft tissue infections worldwide [63–65], and it quickly attains antimicrobial resistance *via* mutation or horizontal transfer of resistance genes from other bacteria [66]. *S. pyogenes* is one of the most vital pathogenic bacteria that cause skin and soft tissue infections worldwide. It can produce infections in the superficial epidermis and keratin layer as well as, in the subcutaneous tissue. Moreover, it is responsible for scarlet fever and streptococcal toxic shock syndrome. [67, 68]. *P. aeruginosa* is one of the most significant pathogens responsible for a wide diversity of hospitalized patients’ infections. It is essentially resistant to various antimicrobial drugs due to its outer membrane’s low permeability and the constitutive expression of several efflux pumps with wide substrates [69]. Also, it is resistant to usually used antiseptics, so it is hard to remove it from infected sites [70]. *C. perfringens* causes tissue necrosis, bacteremia, emphysematous cholecystitis, and gas gangrene [71–73]. Moreover, several previous reports explained the role of *E. coli* in skin and soft tissue infections, and it is considered the leading contributing cause of neonatal omphalitis, lower or upper limb cellulitis, necrotizing fasciitis, surgery site, and after burn injuries infections [74]. In this work, three tests were implemented to estimate the antibacterial activity of the tested EOs, including agar diffusion, broth microdilution, and biofilm formation. It was observed that the gram-negative *P. aeruginosa* and *E. coli*, as well as gram-positive *C. perfringens* were slightly sensitive to HD, MAHD, and SF EOs with IZs ranging from 9-14 mm. On the other side, the Gram-positive *S.aureus* as *S. pyogenes* bacteria displayed resistance to the three oils. These results could be attributed to the fact that although the agar diffusion test accepts that the antibacterial drugs diffuse quickly in the solid nutrient medium [75], this hypothesis, in some cases, results in essential deviations from the expected effects as a result of inconsistency among the volatile compounds during diffusion. Comparing our results with the previous reports, showed that *P. aeruginosa*, *E. coli*, and *S.aureus* were sensitive to HD EOs [23, 32, 36, 76]

Therefore, a microdilution test was applied to give advanced accurateness and overcome the drawbacks of agar diffusion [75, 77]. In this test, MAHD EOs exhibited a significant capability to inhibit the growth of all examined bacterial strains in a dose-dependent manner, followed by HD EOs. Therefore, from the results of the microdilution test, we could conclude that the encouraging antibacterial activity of the investigated EOs, especially that of MAHD, is unnoticeable by the agar diffusion test. The detected antibacterial effect of the investigated oils is highly related to EOs’ chemical components. For instance, the HD and MAHD EOs of *A. *fragrantissima are rich in oxygenated monoterpene ketones as *α*-thujone and its isomer *β*- thujone. It was reported that the thujone compounds and thujone-containing EOs exerted promising antimicrobial activity [78–80]. Also, the two oils contain artemisia alcohol, which is active against different bacterial and fungal strains [80]. Moreover, piperitone was present in major amounts in the three tested essential oils and has been reported to contribute to their antibacterial activity [81].

Additionally, the MAHD EO displayed potent MIC and MBC values against all tested microorganisms and was rich in sesquiterpenes as spathulenol (12.22%) and viridiflorol (7.31%) compared to HD and SF EOs that have been reported to play a significant role as antimicrobial agents [82–84]. Moreover, SF EO is also rich in *β*-Sitosterol (11.07), which is reported to exert antibacterial activity [85]. Additionally, although the investigated EOs showed some similarities in their significant constituents, they exhibited different MIC and MBC, which may be in part due to the difference in each single compound percentage, also the ability of synergism among the major compounds or between the minor and significant compounds [86, 87]. Therefore, synergistic effects between EO compounds may enhance the plasma membrane permeability and their binding to transmembrane proteins, even though the mechanism of the antimicrobial effect is usually relatively complicated to determine [88]. It was reported that the oil rich in ketones ( thujone and, piperitone) as in HD and MAHD EOs possess potent antibacterial activity due to the presence of a carbonyl group which enhances the activity.[89]. Also, the presence of an unsaturation in cyclohexane ring increases the antibacterial activity of pipertone. Moreover, the presence of alcohols (spathulenol and viridiflorol in MADH EO and artemisia alcohol in HD and MAHD EOs) can increase the antibacterial activity as they act by denaturing proteins [89].

It is approved that the phenotypic characteristics of bacteria grown in biofilms significantly distinguish them from those grown in suspension because biofilms are considered the typical environment for most bacteria. In this context, it was reported that EOs exhibited potent biofilm inhibition performed by different mechanisms. For example, they use their hydrophobic nature to modify the cytoplasmic membrane permeability, resulting in the outflow of intracellular content or deactivating the bacteria’s enzymes [90]. Moreover, they might block the quorum-sense system, inhibiting the transcription of the flagellar genes and controlling the motility of the bacteria [91]. In contrast, another report acknowledged the EOs’ ability to reduce bacterial adherence to inert surfaces [92]. Another study reported that EOs improved the reactive oxygen species accumulation and enhanced oxidative stress, leading to cellular apoptosis [93]. Therefore, we aimed to investigate the inhibition perspective of EO on the formation of biofilm.

Interestingly, the results stressed that the SF-EOs positively inhibit Grams’ positive bacteria biofilm more than the negative ones. At the same time, the HD-EOs were more effective in the *P. aeruginosa* biofilm, and MAHD was potent toward *S. pyogenes*. SF-EOs are rich in *β* sitosterols, which exhibited significant antibiofilm activities against pathogenic bacteria [94–97]. At the same time, the HD-EOs were more effective in the *P. aeruginosa* biofilm, and MAHD was potent in affecting *S. pyogenes*. The three oils contain *α*-thujone, which was reported to possess the capability to inhibit the *P. aeruginosa* biofilm[98]. Therefore, we hypothesized that variation in the composition of each EO is a constructive issue that may limit the resistance developments, which is a communal factor for synthetic agents [99].

Inflammation is a communal physiological response. When this response is acute, it can be protective, but when it becomes chronic, it can cause a diversity of ailments [100]. Several types of leukocytes, lymphocytes, and other inflammatory cells are triggered during the inflammation. Macrophages play an essential role in several inflammatory diseases through the induction of proinflammatory mediators [101]. LPS is a potent inducer of monocytes to macrophages, triggering proinflammatory mediators’ production [102]. In the inflammation course, NO, the key proinflammatory mediator, is produced *via* enzyme-inducible iNOS. NO plays an essential role in the immune system. Thus, NO inhibitors can provide exceptional prospects for designing novel therapeutic approaches for inflammatory ailments. It was reported that EOs had been extensively examined as alternative anti-inflammatory agents since the volatile components possess low molecular weight and lipophilic properties that can saturate cell membranes; therefore, they display anti-inflammatory activity in cells [103]. Several reports explained the mechanism by which EOs can inhibit inflammation as they can reduce the mRNA or protein expression of proinflammatory cytokines such as IL-1, IL-6, and TNF-α, so relieving inflammation due to injury; also, they can inhibit COX and LOX activities in the arachidonic acid metabolic pathway as they can decrease the PGE2, therefore, alleviating the inflammatory response. Another mechanism is inhibiting the iNOS and tyrosinase expression or eliminating excessive reactive oxygen species to improve the injury effect of free radicals as well, and they can relieve edema of the tissue and encourage wound healing caused by infection-induced inflammation [104]. Our result revealed that the *A*. *fragrantissima* EOs exhibited significant anti-inflammatory activity by decreasing the release of TNF-α, IL-2, and IL-6 by LPS-activated RAW 264.7 cells and reducing the LPS-induced expression of iNOS. The HD and SF-EO are characterized by the presence of alcohol and ketones such as Santolina alcohol, *α*- and *β*-thujone, and piperitone, which are characterized by the presence of particular OH and C= O that can inhibit inflammation through decreasing the levels of cytokine or by enhancing antioxidant activity [105–107].

Moreover, SF EOs contain a large percentage of *β*-sitosterol, which was previously reported to have anti-inflammatory activity [108]. It can reduce the expression of proinflammatory mediators such as COX-2—TNF-α and iNOS, in LPS-stimulated RAW264.7 macrophages [109]. Furthermore, the synergism between EO compounds may enhance the anti-inflammatory activity of *A*. *fragrantissima.* Despite several EOs being commonly considered safe agents, further studies are required concerning the safety and dosing in a clinical study.

## Conclusions

*A.fragrantissima* aerial parts EOs were prepared using four different techniques, and their components were compared qualitatively and quantitatively. The SF method gives the highest oil yield compared to other methods. *α*-thujone is the main compound in HD and HS EO, while ( +)-spathulenol and piperatone were the major in MAHD and SF EO, respectively HD, MAHD, and SF EOs exhibited susceptibility against *P. Aeruginosa, E. coli* and *C. perfringens*. MAHD EOs demonstrated potent growth inhibition, followed by HD EOs, on all tested microorganisms in the microdilution assay. SF EOs significantly inhibited the biofilm formation of all tested microorganisms, while MAHD and HD -EOs efficiently suppressed *S. pyogenes* and *P. aeruginosa,* respectively. Moreover, HD and Sf EOs exerted potent anti-inflammatory activity by suppressing the TNF-α, IL-2, and IL-6 release and iNOS expression in LPS-stimulated RAW 264.7 macrophages. *A****.**** fragrantissima* EOs comprises different volatile compounds with antibacterial and anti-inflammatory activities that are encouraged to be used as bioactive agents for adjusting skin infections. However, additional studies are essential for their safety in clinical settings.

## Supplementary Information

Supplementary Material 1.

## Data Availability

All data generated or analysed during this study are included in this published article [and its supplementary information files].

## Abbreviations

EOs: Essential oils
HD: Hydrodistillation
MADH: Microwave-assisted hydrodistillation
Hs: Head-space
SF: Supercritical fluid
TNF-α: Tumor necrosis factor-alpha
IL-2, and IL-6: Interleukin-2 and 6
LPS: Lipopolysaccharides
iNOS: Nitric oxide synthesis
IZ: Inhibition zone
GC/MS: Gas Chromatography/Mass spectrometry
MHA: Mueller Hinton agar
MHB: Mueller Hinton broth
DMSO: Dimethyl sulphoxide
MIC: Minimum inhibitory concentration
MBC: Minimum bactericidal concentration
MTT: (3-(4,5-Dimethylthazolk-2-yl)-2,5-diphenyl tetrazolium bromide)ant
ELISA: Enzymes linked immunosorbent assay
SCC: Supercritical carbon dioxide
