# Supplementary material for: Chemical analysis, antibacterial and anti-inflammatory effect of Achillea fragrantissima essential oil growing wild in Egypt
Source: BMC Complement Med Ther. 2024 Nov 7;24:385. doi: 10.1186/s12906-024-04633-9 (PMC11546401; doi:10.1186/s12906-024-04633-9)
Supplement: Supplementary file 1 — Supplementary Material 1. [file 12906_2024_4633_MOESM1_ESM.docx]

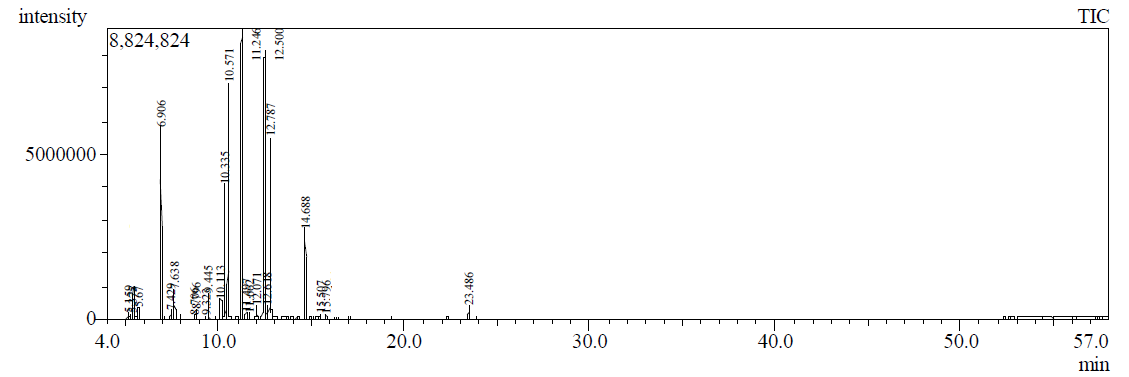


**Fig. S1. GC- MS chromatogram of HS extracted oil**

**
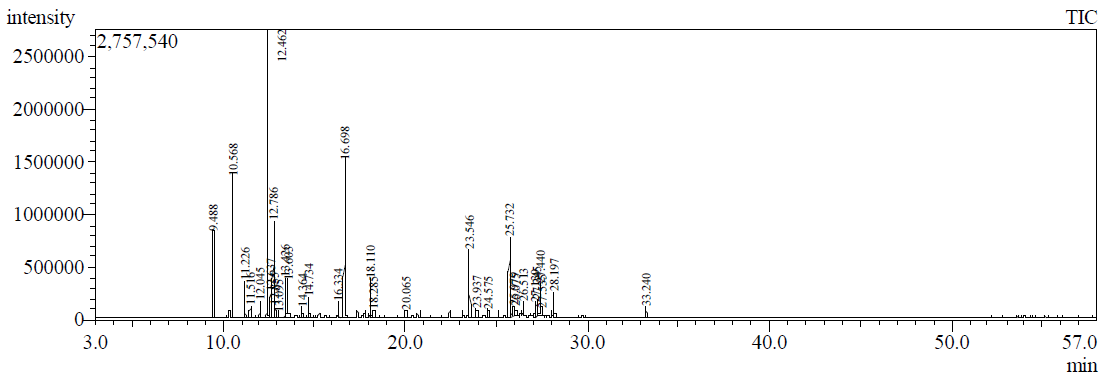
**

**Fig. S2. GC- MS chromatogram of HD extracted oil**

**
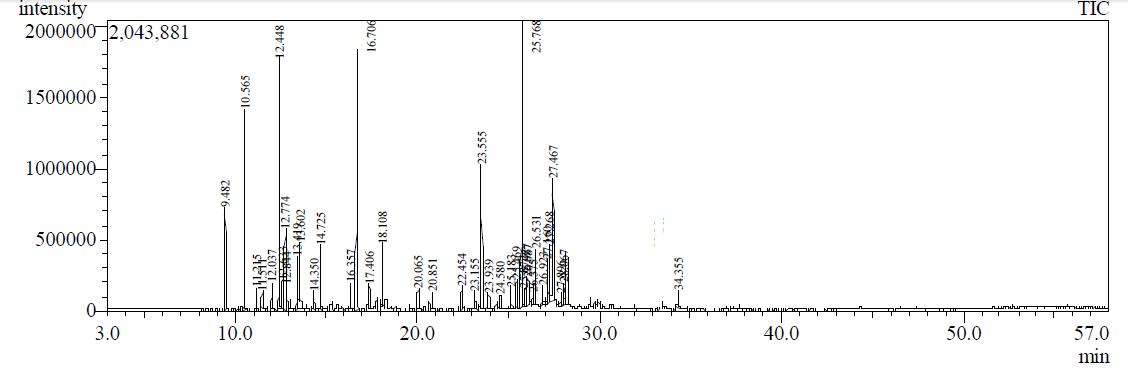
**

**Fig. S3. GC- MS chromatogram of MAHD extracted oil**

**
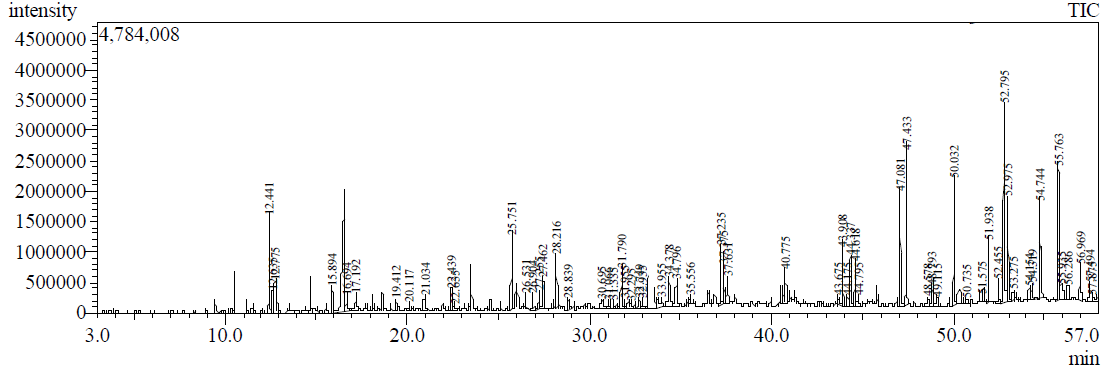
**

**Fig. S4. GC- MS chromatogram of SF extracted oil**

| **B-actin** | **i-NOS** |
| --- | --- |
| 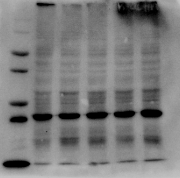 | 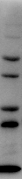  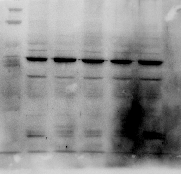 |

**Un-cropped western blot images**
